# Nudging physical distancing behaviors during the pandemic: a field experiment on passengers in the subway stations of shiraz, Iran

**DOI:** 10.1186/s12889-022-13184-y

**Published:** 2022-04-10

**Authors:** Ramin Shiraly, Nika Khoshdel, Ali Khani Jeihooni, Mary-Louise McLaws

**Affiliations:** 1 Department of Community Medicine, School of Medicine, Health Behavior Science Research Center, Shiraz University of Medical Sciences, Shiraz, Iran; 2 Student Research Committee, Shiraz University of Medical Sciences, Shiraz, Iran; 3 Nutrition Research Center, Department of Public Health, School of Health, Shiraz University of Medical Sciences, Shiraz, Iran; 4 School of Population Health, UNSW Sydney, Sydney, Australia

**Keywords:** COVID-19, Pandemic, Physical distancing, Public health, Protection motivation theory, Nudging

## Abstract

**Background:**

The possibility of the emergence of new pandemics necessitates further research into using simple strategies to promote social distancing behaviors in public. Most of the current evidence on effectiveness of physical distancing interventions is based on self-report and measure of intention which will not necessarily predict actual behavior.

**Methods:**

A field experimental study was conducted in the subway stations of Shiraz, Southern Iran. The interventions were based on intuitions from protection motivation theory and consisted of using environmental nudges to notify the passengers of the pandemic situation (threat appeal) and a verbal advice on keeping a safe physical distance as an effective method of protection against COVID-19 (coping message). Average physical distancing was estimated as the number of steps between two consecutive passengers and was compared between interventions (*n* = 1045) and the control (*n* = 855) groups.

**Results:**

A total of 1900 people riding on subway escalators were directly observed during two intervention conditions and the control condition. Under either threat or coping-based interventions, passengers were two times more likely (OR 2.0, 95%CI 1.5–2.7, *P* <  0.001) to keep a physical distance of at least 1.2 m from the traveler in front compared with those who did receive no intervention. The Kruskal-Wallis test revealed that there was a significant improvement in physical distancing behaviors with coping advice compared with threat appeal and the control conditions (χ2 = 120.84, df = 2, *p* <  0.001).

**Conclusions:**

Our findings suggest that simple and inexpensive theory-based interventions can be used in crowded public spaces to promote physical distancing within the context of the pandemic.

## Background

Since December 2019, the COVID-19 pandemic has spread worldwide, killed more than 5 million people and contributed to substantial morbidity and economic losses. The World Health Organization (WHO) recommends key public health strategies to prevent community transmission of SARS-CoV-2: mask use, physical distancing and avoiding crowded low-ventilated environments [1]. Significant practical, motivational and social barriers impede compliance with these public health practices. Encouraging safe physical distancing in the community requires mindfulness in communities who are already stressed from quarantine and lockdown restrictions [2]. As a result, fewer people have been observed complying with the public health measures the longer the pandemic continues [3]. A study on a representative sample of 14 countries as well as mobility and policy data for 124 countries showed that, between March to December 2020, there was a linear rise in mask wearing behaviors but physical distancing compliance declined [4]. Despite global mass vaccination efforts, reduced adherence to social distancing measures in high-risk settings may lead to further mutations of SARS-CoV-2 and create new waves of outbreaks and squander the previous public health efforts made by international, national and local health organizations to control the pandemic [5].

The WHO has acknowledged that SARS-CoV-2 is predominantly transmitted by direct or indirect close contact with infected persons or their respiratory droplets that are exhaled during sneezing, coughing and talking, however, the transmission via fomites is likely and transmission via aerosols is possible in indoor crowded spaces [6]. Physical distancing is one of the most effective measures to reduce the spread of respiratory viruses [7]. It has been demonstrated that in both health-care and community settings, physical distancing of 1 m or more is associated with significant reduction in risk of infection with SARS-CoV-2 [8, 9].

There has been a diverse range of interventions implemented across the world to promote compliance with physical distancing such as restrictions and education. Most of the current evidence on effectiveness of physical distancing interventions is based on self-report and measure of intention which will not necessarily predict actual behavior [10, 11]. In the present research, a novel way was used to quantitatively estimate physical distances in certain public situations when people are expected to be able to maintain an adequate distance from others. We designed a field experimental study to observe whether simple interventions in subway stations can trigger safer physical distancing behaviors. The main assumption of the current study is that people behave more safely during the pandemic if their recognition of the threat is enhanced (threat appraisal) or they are informed about the appropriate protective responses to take (coping appraisal).

The interventions applied consisted of a threat appeal (environmental nudges were used to warn people about the risks of the pandemic) and a coping message (verbal persuasion was used to influence people’s physical distancing behavior). Our interventions were based on intuitions from protection motivation theory.

## Protection motivation theory

Protection Motivation Theory (PMT) aims to explicate the cognitive processes that regulate people’s behavior in the context of a threat or hazard [12]. When people face a threatening situation, they tend to evaluate the significance of the threat identified and anticipate probable consequences of different actions aiming to eliminate the threat or reduce it to an acceptable level. The decisions then taken are based on adaptive and maladaptive responses as a consequence of two appraisal processes: threat appraisal and coping appraisal. Threat appraisal refers to people’s evaluation of the degree to which an event has significant implications for their well-being. Threat appraisal consists of perceived vulnerability (an individual’s estimation of the chance of experiencing the threat) and perceived severity (an individual’s estimation of the seriousness of a threat and its consequences). Coping appraisal refers to the judgment of one’s capability to cope with the threat and an assessment of how much a threat can be reduced or eliminated if a certain behavior is performed. Coping appraisal consists of self-efficacy (the belief in one’s ability to execute the recommended courses of action successfully), response efficacy (an individual’s expectation that carrying out the recommended action will remove the threat), and response costs (the costs associated with practice of the recommended behavior) [13].

Accordingly, we postulated the following hypotheses:

- H1: Enhanced perceived threat via environmental nudges improves physical distancing behavior.
- H2: Enhanced perceived coping ability via verbal persuasion improves physical distancing behavior.

To enhance perceived threat regarding the pandemic, we planned to use environmental nudges as a behavioral intervention to draw people’s attention to the pandemic situation and notify them about the risk of COVID-19 infection. Nudges are subtle environmental cues that guide choices without restricting them and when applied, moves individuals towards rational behavioral patterns [14].

A large body of research has demonstrated that using theory-based interventions will improve the effectiveness of behavior change interventions [15]. It has been shown that protection motivation theory can be a useful framework for understanding intention to engage in social distancing behavior [16].

Considering the unique timeframe of one of history’s most deadly pandemics and uncertainties about how best to promote people to learn and accept physical distance as a social normative behavior, we aimed to assess the effect of two simple and inexpensive theory-based interventions including environmental nudges and verbal persuasion to move passengers towards safer physical distancing in subway stations.

## Methods

### Study design and participants

We conducted a field experimental study of the response to simple threat and coping-based interventions to encourage people to comply with physical distancing in crowded subway stations of Shiraz, a metropolitan city in Southern Iran with a population of approximately two million. Four subway stations with similar levels of crowding in the inner city of Shiraz were selected for the study. We measured physical distances in queues as people entered escalators during peak times, 8:00 AM to 10:00 AM, in the selected subway stations between January 5 and January 13, 2021. Inclusion criteria for observations were individuals who were travelling on ascending or descending escalators and had someone in front when stepping up or down the escalator and the person was judged to be able keep their distance from the person in front. Distances between people who were walking together, such as friends or families were excluded from the observations. Observers made judgments of the observed passengers’ age group (young, middle aged, elders) and gender (male, female) and whether they wore a face mask. We used a convenient sample of observations during control (no intervention) and two intervention conditions.

### Interventions

Two types of interventions were used: environmental nudges as threat appeal and verbal advice as coping message. We used environmental nudges to notify the risk of contracting COVID-19 infection. In the threat appeal condition, there were three staff at the site who wore protective clothing and face mask and shield and overtly clean touch surfaces such as the escalator handrails with disinfectants. They also offered alcohol-based hand sanitizer to passengers who wanted to disinfect their hands while waiting in queue and before they stepped onto the escalator. During this experiment, no verbal communication including educational information was given to the passengers. In the coping message condition, verbal advice was given by personnel respectfully requesting passengers to keep an adequate physical distance as an effective method to protect against COVID-19, before entering the escalators. In this condition, personnel just wore face mask without any additional protective equipment. Both interventions including verbal advice and environmental nudges were used to trigger passengers to practice a COVID-19 public health measure of physical distancing. It was assumed that environmental nudges increased both the perceived threat and the perceived vulnerability about the risk of contracting COVID-19 infection and the verbal advice enhanced both the response efficacy and the self-efficacy to cope with the risk of infection.

### Measurements

Physical distancing was defined as the sum of the number of steps between the target (observed) passenger and the person in front on the escalator. Each escalator step was measured 40 cm (0.4 m) in depth; the depth of the escalator steps was the same in all four stations. Physical distances were estimated as the sum of the number of steps between two consecutive passengers while they were in a stable position on the escalators. Responses to three approaches were examined: observations of no intervention and observations during two intervention conditions. Observations were concurrently recorded by two observers and our findings revealed nearly perfect inter-observer agreement for all the observations performed. The data collection process was supervised by an experienced member of the team research. Throughout the study it was made sure that the personnel maintained a safe physical distance while standing on the side or working at the place and did not obstruct passengers. Observers recorded the physical distances between passengers when they set foot onto the escalator and stood at a stable position after not receiving an intervention or after having received one of the two interventions. Passengers were considered as having maintained an adequate physical distance from each other when observed to deliberately space themselves by at least three steps from the person in front of them on the escalator. Observed physical distances were classified as safe and unsafe behaviors: keeping a physical distance of at least three steps (≥ 1.2 m) was considered as safe and a distance of two steps or lower (< 1.2 m) was considered as unsafe physical distancing behavior. These two behavior classifications were compared between threat and coping based interventions and the control group.

### Statistical analysis

Descriptive statistics, including frequency (%) and means ± standard deviation (SD) were calculated for observational data. Associations between interventions and control conditions and safety of physical distances were assessed using a chi-squared test for significance, and an odds ratio (OR) and confidence intervals (95%CI) were calculated. Normality was checked using the Shapiro-Wilk test and by visual inspection of normality plots. Due to non-normal distribution of the data, the Kruskal-Wallis test was used to compare physical distances in three different conditions. Multivariate logistic regression was used to identify independent factors associated with safe physical distancing behavior. Data were analyzed using SPSS (Version 23, SPSS Inc., USA). A *p*-value of less than 0.05 was considered statistically significant.

## Results

We made 1900 direct observations. There were 855 (45%) observations during control (no intervention) condition, 370 (19.5%) during coping-based intervention via verbal advice, and 675 (35.5%) during threat appeal condition. The descriptive statistics of observed passengers in these three conditions are given in Table 1. Over half of the passengers (1066/1900, 56%) were male. Over half were young (1116/1900, 59%), while 30% (573/1900) were assessed as middle-aged and 11% (211/1900) were older adults. Face masks were worn by 98% of the observed passengers. Mean physical distance in steps was 1.25 ± 1.03 (range: 0–6, mode: 1). The Kruskal-Wallis test revealed that there was a statistically significant difference in physical distancing between the two interventions and the control conditions (χ2 = 120.84, df = 2, *p* <  0.001), indicating a significant improvement in physical distancing behaviors with verbal advice compared with environmental nudges and the control conditions. The mean ranks of physical distance with verbal advice, environmental nudging and no intervention were 1090.1, 1058.0 and 805.2, respectively. Table 2 compares the likelihood of keeping a safe physical distance during the interventions and the control conditions. Overall, 88.4% of observed physical distances were assessed as unsafe (less than 1.2 m distance). There was a significant difference in physical distancing between those who received any of two interventions compared with no intervention. Under intervention conditions, passengers were two times more likely (OR 2.0, 95% CI 1.5–2.7, *P* <  0.001) to keep a safe distance of 1.2 m or more from the traveler in front compared with those who did receive no intervention. When verbal advice was used, passengers were 2.6 times more likely (OR 2.6, 95% CI 1.8–3.7, *P* < 0.001) to keep a safe distance of 1.2 m or more from other passengers compared with those who did not receive any intervention. Interestingly, coping-based intervention through verbal advice was more influential compared with threat-based intervention via environmental nudging (OR 1.5, 95% CI 1.1–2.1, = 0.022) (Table 2). Safe physical distancing was not associated with gender (*P* > 0.10) but the middle-aged and elderly passengers kept a greater physical distance than younger individuals (*P* = 0.001).

**Table 1:** Descriptive characteristics of observed passengers in the control and intervention conditions

Variables	No intervention (*n* = 855)	Coping message (*n* = 370)	Threat appeal (*n* = 675)	Total (*n* = 1900)
Gender (n, %)				
Men	489 (57)	213 (58)	364 (54)	1066 (56)
Women	366 (43)	157 (43)	311 (46)	834 (44)
Age group (n, %)				
Young	483 (57)	239 (65)	394 (58)	1116 (59)
Middle-aged	274 (32)	109 (30)	190 (28)	573 (30)
Older	98 (11)	22 (6)	91 (14)	211 (11)
Mask wearing (n, %)				
Yes	835 (98)	359 (97)	665 (98)	1859 (98)
No	20 (2)	11 (3)	10 (2)	41 (2)
Physical distance (Mean ± SD)				
Step	1 ± 1	1.5 ± 1	1.4 ± 1	1.3 ± 1
Meter	0.40 ± 0.40	0.62 ± 0.44	0.57 ± 0.40	0.50 ± 0.41

**Table 2:** Comparison of keeping a safe physical distance between travelers in different control and intervention conditions

Type of intervention	Physical distances, N (%)		OR (CI 95%)	*P* value
	Safe (≥ 1.2 m)	Unsafe (< 1.2 m)		
Threat appeal / Coping message	153 (15)	892 (85)	2.0 (1.5–2.7)	< 0.001
Control (no-intervention)	67 (8)	788 (92)		
Coping message	67 (18)	303 (82)	2.6 (1.8–3.7)	< 0.001
Control (no-intervention)	67 (8)	788 (92)		
Threat appeal	86 (13)	589 (87)	1.7 (1.2–2.4)	0.002
Control (no-intervention)	67 (8)	788 (92)		
Coping message	67 (18)	303 (82)	1.5 (1.1–2.1)	0.022
Threat appeal	86 (13)	589 (87)		

Results of both univariate and multivariate regression analysis of independent variables showed that threat and coping-based interventions (environmental nudges and verbal advice) were positively associated with keeping a safe physical distance of at least 1.2 m (OR = 2.07, 95% CI: 1.53 to 2.81, *P* < 0.001). A younger age was negatively associated with a safe distancing behavior (OR = 0.63, 95% CI: 0.47 to 0.83, *P* = 0.001). There was no statistically significant association between gender and mask wearing status of the passengers with physical distancing (Table 3).

**Table 3:** Variables associated with keeping a safe physical distance (≥1.2 m) between travelers riding on subway escalators

Variables	Univariate		Multivariate	
	OR (95% CI)	***P*** value	OR (95% CI)	***P*** value
Young age	0.66 (0.49 to 0.87)	0.003	0.63 (0.47 to 0.83)	0.001
Older age	1(ref)		1 (ref)	
Female	1.05 (0.79 to 1.40)	0.711	1.08 (0.81 to 1.44)	0.595
Male	1 (ref)		1 (ref)	
Mask wearing	1.88 (0.86 to 4.10)	0.114	2.01 (0.93 to 4.60)	0.078
No mask wearing	1 (ref)		1 (ref)	
Threat/coping interventions	2.01 (1.49 to 2.73)	< 0.001	2.07 (1.53 to 2.81)	< 0.001
No-intervention	1 (ref)		1 (ref)	

## Discussion

Our study demonstrated that passengers who were exposed to either threat appeal or coping message behaved more safely than passengers in the no-intervention condition. These findings suggest that both threat- and coping-based interventions can promote physical distancing in crowded public spaces during the pandemic. The applied interventions were easy to implement and can lead to positive changes in passengers’ social distancing behavior. Previous studies have demonstrated the positive impact of increased threat and coping appraisal on protective behaviors during the pandemics. However, most of these studies were based on hypothetical scenarios. A study conducted in Sweden demonstrated that coping appraisal was associated with improvement in self-reported social distancing behavior during influenza pandemic [17]. A web-based survey which evaluated the effect of PMT components on behavioral responses to pandemic flu found that both threat and coping appraisal were associated with social distancing behaviors, nevertheless, coping component was the principal predictor of how people may behave during pandemics [18]. On the other hand, most published experimental studies about the effect of nudges on compliance with COVID-19 protective measures have used online interventions and text messages [19]. To the best of our knowledge, this is the first study to assess the effect of threat and coping-based interventions on actual physical distancing behavior in a public transport setting during the pandemic. Our findings provides evidence that simple interventions including environmental nudges and verbal persuasion can promote safe physical distancing behavior in the context of the pandemic. Another important finding of the present study is that supporting response efficacy and self-efficacy through verbal coping message has a more powerful influence on physical distancing behavior than enhancing perceived threat through environmental nudges. This finding is in parallel with previous studies which have shown that coping information in order to increase perceptions of response effectiveness and particularly self-efficacy is more important determinant of behavioral change than presenting threatening information in order to increase perceived risk [20]. During the threat appeal condition, we tried to make changes in the subway station environment in order to increase perceived threat and to warn people about the risk of contracting the COVID-19 infection. Seeing staff in protective clothing while disinfecting touch surfaces or offering hand sanitizers was used as a message to increase the levels of fear arousal and to influence people’ awareness of the pandemic situation. In coping message condition, the passengers were simply advised to keep an adequate physical distance as an effective method for reducing the risk of COVID-19 infection. Both of these behavioral interventions seem to be effective, however, our findings revealed that the coping appraisal component of PMT is more efficacious in changing social distancing behavior.

Also, our results showed that older and middle-aged passengers were more likely to keep a safe physical distance than younger individuals. This may be due to the fact that middle-aged and particularly the older adults are more susceptible to COVID-19 complications and subsequent hospitalizations [21]. Fear of infection and subsequent death may affect this age group’s intention to engage in and adopt preventive measures such as physical distancing.

We chose subway stations as the study setting because in the pandemic situation, crowded public places in metropolitan areas can pose a significant risk for COVID-19 transmission and adherence to social distancing measures is necessary. Overcrowded low-ventilated vehicles and stations can facilitate the transmission of the respiratory infections, particularly during peak times when keeping a safe physical distance might be much more difficult [22, 23].

Investigation of the effect of interventions on physical distancing behaviors is restricted by methodological issues and most related studies are based on self-report rather than actual behavior [11]. In the present study, we used a practical methodology to influence and estimate physical distancing in public. Direct observation is a superior method compared to self-report and allowed us to quantitatively assess physical distances between individuals on subway escalators.

Community interventions aimed at physical distancing must be acceptable, effective, and sustainable. Traditional policies to change health behaviors mostly focus on education, legislation, and regulation. Most of the evidence that postulates effectiveness of physical distancing interventions is derived from modelling and self-report studies or examining restrictions such as mass gatherings, closure of schools and workplaces, public transport and lockdown [10]. Our findings support the ease of assisting the community to achieve safer behavior through simple techniques that may be that pivotal for success. We believe that simple behavioral interventions, particularly the coping-based interventions, can positively influence the public’s behavior to distance and engage normalizing protective behavior during the pandemic.

### Limitations

There are some limitations to this study that should be noted. At the time of the study, face masks were mandatory on Iran subways, and as a result, in such study setting, there might be an insufficient number of mask non-wearers to accurately assess the association between wearing face masks and keeping physical distances. Additionally, we did not evaluate touching handrails by passengers as it could depend on other factors such as keeping balance while walking onto the escalator. Another limitation of the study was the removal of observations of some passengers whose movements were abrupt and unexpected moved back and forth when stepping on the escalator that altered the physical distance. Maintaining a distance of at least 1.8 m distance from others has been recommended by the US Centers for Disease Control and Prevention to protect against COVID-19 infection [24]. These distances were unlikely to be seen in overcrowded subways of the present study setting and we attempted to encourage at least a three step-distance (1.2 m) between passengers. We did not evaluate the effect of implementing both coping messaging and warning nudges at the same time to understand whether there is any additional effect.

## Conclusions

Both environmental nudges and verbal persuasion could promote physical distancing by the public during a pandemic. Supporting coping appraisal through verbal advice has a more powerful influence on physical distancing behavior than enhancing perceived threat by environmental changes. As these simple interventions are inexpensive to execute and can benefit passengers during public transport, these approaches are cost-effective tools that could be used by public health organizations within the context of the pandemic.

## Data Availability

The datasets generated and/or analyzed during the current study are not publicly available due to due to institutional regulations and privacy restrictions but are available from the corresponding author upon reasonable request.

## Abbreviations

PMT: Protection motivation theory
CI: Confidence interval
W.H.O: World Health Organization
